# The associations between type 2 diabetes and plasma biomarkers of Alzheimer’s disease in the Health and Aging Brain Study: Health Disparities (HABS-HD)

**DOI:** 10.1371/journal.pone.0295749

**Published:** 2024-04-01

**Authors:** Fang Yu, Keenan A. Pituch, Molly Maxfield, Elsa Baena, Yonas E. Geda, Jeremy J. Pruzin, David W. Coon, Gabriel Q. Shaibi, HABS-HD Study Team

**Affiliations:** 1 Edson College of Nursing and Health Innovation, Arizona State University, Phoenix, Arizona, United States of America; 2 Clinical Neuropsychology Department, Barrow Neurological Institute, Phoenix, Arizona, United States of America; 3 Department of Neurology and the Franke Neursciene Education Center, Barrow Neurological Institute, Phoenix, Arizona, United States of America; 4 Department of Neurology, Banner Alzheimer’s Institute, Phoenix, Arizona, United States of America; Instituto Nacional de Geriatria, MEXICO

## Abstract

Alzheimer’s disease (AD) affects Latinos disproportionately. One of the reasons underlying this disparity may be type 2 diabetes (T2D) that is a risk factor for AD. The purpose of this study was to examine the associations of T2D and AD blood biomarkers and the differences in these associations between Mexican Americans and non-Hispanic Whites. This study was a secondary analysis of baseline data from the observational Health and Aging Brain Study: Health Disparities (HABS-HD) that investigated factors underlying health disparities in AD in Mexican Americans in comparison to non-Hispanic Whites. HABS-HD participants were excluded if they had missing data or were large outliers (z-scores >|4|) on a given AD biomarker. Fasting blood glucose and glycosylated hemoglobin (HbA1c) levels were measured from clinical labs. T2D was diagnosed by licensed clinicians. Plasma amyloid-beta 42 and 40 (Aβ_42/42_) ratio, total tau (t-tau), and neurofilament light (NfL) were measured via ultra-sensitive Simoa assays. The sample sizes were 1,552 for Aβ_42/40_ ratio, 1,570 for t-tau, and 1,553 for NfL. Mexican Americans were younger (66.6±8.7 vs. 69.5±8.6) and had more female (64.9% female vs. 55.1%) and fewer years of schooling (9.5±4.6 vs. 15.6±2.5) than non-Hispanic Whites. Mexican Americans differed significantly from non-Hispanic Whites in blood glucose (113.5±36.6 vs. 99.2±17.0) and HbA1c (6.33±1.4 vs. 5.51±0.6) levels, T2D diagnosis (35.3% vs. 11.1%), as well as blood Aβ_42/40_ ratio (.051±.012 vs. .047±.011), t-tau (2.56±.95 vs. 2.33±.90), and NfL levels (16.3±9.5 vs. 20.3±10.3). Blood glucose, blood HbA1c, and T2D diagnosis were not related to Aβ_42/40_ ratio and t-tau but explained 3.7% of the variation in NfL (*p* < .001). Blood glucose and T2D diagnosis were not, while HbA1c was positively (*b* = 2.31, *p* < .001, *β =* 0.26), associated with NfL among Mexican Americans. In contrast, blood glucose, HbA1c, and T2D diagnosis were negatively (*b =* -0.09, *p* < .01, *β =* -0.26), not (*b =* 0.34, *p* = .71, *β =* 0.04), and positively (*b* = 3.32, *p* < .01, *β =* 0.33) associated with NfL, respectively in non-Hispanic Whites. To conclude, blood glucose and HbA1c levels and T2D diagnosis are associated with plasma NfL levels, but not plasma Aβ and t-tau levels. These associations differ in an ethnicity-specific manner and need to be further studied as a potential mechanism underlying AD disparities.

## Introduction

Type 2 diabetes (T2D) and Alzheimer’s disease (AD) are among the most common, costly, and disabling diseases globally [1]. T2D is characterized by chronic insulin resistance and hyperglycemia [2] while AD pathologies include Amyloid-beta plaques, Tau tangles, and Neurodegeneration (ATN) in the brain [3]. Despite their seemingly different features, T2D and AD are crosslinked by insulin resistance and hyperglycemia [2, 4–10], inflammation, and oxidative stress [3, 4, 6, 11–18]. Many T2D animal studies support that T2D precedes AD [19] and hyperinsulinemia and hyperglycemia induce Aβ overproduction and cognitive decline [6, 20–22]. Furthermore, pharmacologic therapies used to treat T2D show some promise for reducing ATN [23–28].

In humans, abnormal insulin signaling was first reported in postmortem brain tissue of individuals with AD [4, 5]. Fluorodeoxyglucose Positron Emission Tomography (FDG-PET) of the brain show that adults with normal cognition but at risk or with T2D experienced regional cortical hypometabolism that is frequently implicated in AD [29–31]. Hyperglycemia is associated with cerebral amyloid burden [32] and AD clinical progression [19, 33]. Impaired insulin signaling is further associated with PET amyloid burden and cerebrospinal fluid (CSF) biomarkers of AD, hyperphosphorylated tau 181 (p-tau181) and Aβ_42/40_ ratio [34]. T2D and higher glycosylated hemoglobin A1c (HbA1c) have been associated with the neurodegeneration characteristics of AD [30, 35, 36]. Further, impaired fasting glucose is associated with increased cerebral amyloid [32] and accelerates AD clinical progression [19, 33]. HbA1c, longer T2D duration, poorer glycemic control, and diabetic complications are associated with more cognitive impairment [19]. In contrast, other studies did not find an association between T2D or HbA1c with CSF Aβ_42_ [30]. T2D or its duration was not found to affect memory in individuals with mild cognitive impairment (MCI) and AD [37]. Some exploratory analyses even suggest that comorbid T2D might be cognitively and functionally protective in older adults with mild AD dementia [38, 39]. Nonetheless, studies examining T2D and AD are limited with mixed findings due to large variations in methods and the clinical phase of AD under study [30, 40]. The cost and invasiveness of measuring PET and CSF AD biomarkers can now be somewhat overcome with plasma ATN biomarkers [41–43].

Furthermore, T2D and AD are more prevalent in Hispanic Americans with T2D affecting 22.6% of Hispanics (vs. 11.3% of non-Hispanic Whites) and AD afflicting 14–21% of Hispanics (vs. 10% in Whites) [44–47]. Hispanic Americans also experience AD at a younger age of onset of AD, longer disease duration, and worse cognition proximal to death than other ethnic groups [46]. Despite the disproportionate burden of AD on Mexican Americans, they have been underrepresented in AD research.

The purpose of this study was to examine the associations of T2D and AD plasma biomarkers and differences in these associations among Mexican Americans in comparison to non-Hispanic Whites. In other words, we were studying if the pathological blood markers of T2D and AD are associated among individuals who are cognitively normal because the pathological changes of T2D and AD can take years or even decades to accumulate without producing any symptoms and may be detectable via blood biomarkers in individuals with normal cognition. We hypothesized that: 1) higher blood HbA1c and glucose levels as well as the presence ofT2D diagnosis would be associated with lower plasma Aβ_42/40_ ratio and higher plasma t-tau and neurofilament light (NfL) levels; and 2) the relationships of blood HbA1c and glucose levels as well as the presence of T2D diagnosis with AD plasma biomarkers would be stronger in Mexican Americans than non-Hispanic Whites.

## Materials and methods

### Design

This study was a secondary analysis of baseline data from the Aging Brain Study: Health Disparities (HABS-HD). The purpose of the HABS-HD, an observational study, was to investigate long-term factors underlying health disparities and differential pathways in incident MCI and AD in Mexican Americans in comparison to non-Hispanic Whites. It enrolled 2076 representative participants (1039 Mexican Americans, 1037 non-Hispanic Whites) at baseline from September 2017 to December 2021. During baseline data collection which spanned over 4 months per person, the participant underwent physical exam, functional and cognitive assessment, blood draws, and neuroimaging. A detailed HABS-HD protocol was published previously [48]. The current study was a secondary analysis of de-identified HABS-HD data. The Institutional Review Board at Arizona State University (ASU) considered this study non-human research; hence, waived the requirement for informed consent and exempted the study (ID STUDY00015500).

### Sample

A community-based participatory research approach was used to recruit participants in the HABS-HD. Multi-pronged recruitment strategies were implemented, including community presentations and educational events, newspaper, television, and radio advertisements, social media campaigns, and referrals. Inclusion criteria included self-reported identification as Mexican American or non-Hispanic White, agreement to blood collections, capacity of participating in neuroimaging, 50 years old or older, and fluent in English or Spanish. Exclusion criteria were type 1 diabetes, active infection, current/recent cancer except for skin cancer, current severe mental illness that could impact cognition except for depression, recent traumatic brain injury with loss of consciousness, current/recent alcohol/substance abuse, active severe medical conditions that could impact cognition, and current diagnosis of non-AD dementia [48].

For this study, HABS-HD participants were excluded if they had missing data or were outliers (z-scores >|4|) on a given AD biomarker (Aβ_42/40_ ratio, t-tau, or NfL) within each ethnic cohort. Of the 2076 HABS-HD participants, 524 (25.2%) participants were excluded for Aβ_42/40_ ratio, 506 (24.4%) for t-tau, and 523 (25.2%) for NfL. The analytic sample sizes were then 1,552 for Aβ_42/40_ ratio, 1,570 for t-tau, and 1,553 for NfL.

### Setting

Most data collection for the HABS-HD occurred at the Institute for Translational Research at the University of North Texas Health Science Center. Blood collections for fasting blood and clinical labs took place at Quest Laboratories [48]. Deidentified data were shared with the corresponding author of the current study through the portal of Institute for Translational Research. All data analyses for this study were performed at ASU.

### Variables and their measures

#### Independent variables

Blood glucose and HbA1c levels were obtained from clinical labs. Fasting blood samples were collected and processed according to the international guideline [49]. T2D diagnosis was determined by a licensed clinician (MD, DO, or NP) based on medical history, objective measures, clinical labs, and medications in the HABS-HD. Ethnicity was categorized as Mexican American or non-Hispanic White [48].

#### Dependent variables

A custom automated StarPlus system (Hamilton Robotics) was used to complete assay preparation. Plasma samples were assayed to measure Aβ_42_, Aβ_40_, t-tau, and NfL using the ultra-sensitive Simoa (single molecule array) technology platform (Quanterix.com) based on previously established methods with coefficients of variations for all assays were ≤5% [48, 50]. The Aβ_42/40_ ratio was calculated by dividing Aβ_42_ concentration by Aβ_40_ concentration.

#### Potential covariates

Potential sociodemographic covariates included age, sex, education, marital status, income, homeownership, years living in the U.S., and smoking. Potential clinical covariates were *APOE*4 positivity defined as the presence of at least one E4 allele, cognition measured by Mini-Mental State Examination, health status measured by self-report, depressive symptoms measured by the 30-item Geriatric Depression Scale (GDS) [51], body mass index (BMI), abdominal circumference in inches. Research medical (hypertension, dyslipidemia, cardiovascular decease [CVD], anemia, and hypothyroidism) and cognitive diagnoses (mild cognitive impairment and dementia) were assigned by a study licensed clinician (MD, DO, or NP) based on collected data, including medical history, objective measures, clinical labs, and medications, and neuropsychological test results according to published criteria [48].

### Power and data analysis plan

Given an alpha of .01, that the other predictors in the model account for 20% of the outcome variation, and that a given focal predictor accounts for a small proportion of outcome variation (i.e., ΔR^2^ = .01), *N* of 927 would provide power >.80 to detect the effect of a focal predictor. The analytic sample sizes were then 1,552 for Aβ_42/40_ ratio, 1,570 for t-tau, and 1,553 for NfL.

To describe the sample and examine associations between each study variable and ethnicity, we obtained descriptive statistics by ethnicity and conducted bivariate statistical tests (i.e., Welch’s independent samples *t* test and Fisher’s exact test of association) in SPSS.

To test the study hypotheses, regression analyses were conducted separately for Aβ_42/40_ ratio, t-tau, and NfL, with the same set of predictors included for each outcome. For hypothesis 1, the focal predictors were blood glucose, diabetes diagnosis (coded as 1 = positive; 0 = negative) and HbA1c. Demographic covariates included in the regression models were ethnicity (1 = Mexican American; 0 = non-Hispanic white), age, sex (1 = female; 0 = male), education, marital status (1 = married; 0 = not married), homeowner (1 = homeowner; 0 = otherwise), and number of years living in the U.S. Other covariates were *APOE*4 positivity (1 = yes; 0 = no), MMSE, health status, GDS-30, BMI, and abdominal circumference. Diagnosis variables (each coded 1 = condition is present; 0 = condition is absent) included hypertension, CVD, anemia, hypothyroidism, mild cognitive impairment, and dementia. The regression models for hypothesis 2 had these same predictors but also include the product terms glucose × ethnicity, HbA1c × ethnicity, and diabetes × ethnicity, which were needed to test two-way interactions of the focal predictors by ethnicity. Values of income were divided by 10,000 and values of Aβ_42/40_ ratio were multiplied by 100 to reduce the number of leading zeros in the regression coefficient estimates. The variance inflation factor indicated that excessive multicollinearity was not present, as each variance inflation factor < 5.

Although we excluded cases having missing data for each outcome, the remaining analytic sample had > 100 cases with incomplete data on one or more predictors. Given that exclusion of cases with complete data on an outcome but missing on predictors can lead to biased parameter estimates [52], we obtained regression model parameters using Bayesian Markov Chain Monte Carlo (MCMC) estimation to treat this missing data. This Bayesian procedure (a) yields unbiased parameter estimates and accurate standard error “equivalents” (defined as the standard deviations of the posterior distributions) when data are missing at random and (b) does not require that data meet distributional assumptions, such as normality [52–54]. We monitored model convergence with the potential scale reduction factor [55] with a value less than 1.05 indicating convergence. Bayesian analysis was conducted with Mplus software [56].

Unlike traditional analyses, Bayesian estimation produces a distribution of values for each model parameter, and we requested 10,000 random draws to build these posterior distributions (after 10,000 burn-in iterations). The median of these posterior distributions was used to represent final parameter estimates (e.g., regression coefficients). Further, we obtained one-tailed *p* values based on the posterior distributions of the regression coefficients but doubled these values to compare them to alpha of .05, commonly reported in traditional inference. To convey the practical importance, or meaningfulness, of the analysis results, we obtained raw score (*b*) and standardized regression coefficients (*β*), model *R*^*2*^, as well as the incremental proportion of explained variance (i.e., Δ*R*^*2*^), the latter for the set of focal predictors (for hypothesis 1) and the set of two-way interactions (for hypothesis 2). Note that to obtain the incremental Δ*R*^*2*^ values, we estimated and reported the results for three regression models for each outcome, with the first model excluding the focal predictors and their interaction terms, the second model adding the focal predictors, and the third model adding the set of two-way interactions. Wald tests were used to assess the significance of the model and incremental *R*^*2*^ estimates. For significant interactions involving a continuous focal predictor, the Johnson-Neyman technique [57] was used to identify significance regions where outcome differences between Mexican Americans and non-Hispanic Whites were statistically significant, as determined with 95% Bayesian highest density credibility bands. We graphed significant interactions with SAS software, version 9.4 M7.

## Results

### Participant characteristics

Table 1 displays statistics for the sample by ethnicity. Compared to the non-Hispanic White sample, the Mexican American sample was younger, had less education and income, and lived in the U.S. for fewer years, with a greater proportion of women, and a smaller proportion of homeowners. The Mexican American sample also has greater proportions of current smokers and diagnoses of T2D, hypertension, anemia, mild cognitive impairment, and dementia, a smaller proportion of those with APOE4 positivity, lower MMSE and self-rated health, as well as greater GDS, BMI, and Ab circumference, blood glucose and HbA1c, Ab_42/40_ ratio, t-tau, and NfL.

**Table 1:** Characteristics of the sample by ethnicity.

Variable	*N*	Mean (SD) or Number (Percent)			*p* ^a^
		Overall	Mexican American	Non-Hispanic White	
Age (years)	1,570	66.6 (8.7)	64.0 (8.0)	69.5 (8.6)	< .001
Biological sex	1,570				< .001
Male		623 (39.7)	291 (35.1)	332 (44.9)	
Female		947 (60.3)	539 (64.9)	408 (55.1	
Education (years)	1,570	12.4 (4.8)	9.5 (4.6)	15.6 (2.5)	< .001
Marital status	1,569				.324
Married		964 (61.5)	500 (60.2)	464 (62.8)	
Not married		605 (38.6)	330 (39.8)	275 (37.2)	
Income	1,526	57,852 (56,271)	34,595 (32,724)	83,614 (65,002)	< .001
Homeowner	1,561				< .001
Yes		1,184 (75.8)	597 (72.3)	587 (79.9)	
No		377 (24.2)	229 (27.7)	148 (20.1)	
Years living in U.S.	1,529	55.2 (20.5)	43.3 (20.1)	69.0 (9.1)	< .001
Smoking currently	1,569				.002
Yes		89 (5.7)	61 (7.4)	28 (3.8)	
No		1,480 (94.3)	768 (92.6)	712 (96.2)	
*APOE*4 positivity	1,565				< .001
Yes		370 (23.6)	149 (18.0)	221 (29.9)	
No		1,195 (76.4)	677 (82.0)	518 (70.1)	
MMSE	1,569	27.4 (3.0)	26.1 (3.5)	28.9 (1.4)	< .001
Health status	1,569	2.6 (1.0)	1.9 (1.0)	2.8 (0.8)	< .001
GDS	1,568	5.5 (5.8)	6.5 (6.3)	4.4 (4.8)	< .001
BMI	1,563	29.8 (5.9)	30.8 (5.8)	28.8 (5.7)	< .001
Ab circumference	1,567	39.7 (5.6)	40.2 (5.3)	39.3 (5.9)	.002
Diagnosis					
Hypertension	1,570				.004
Present		984 (62.7)	548 (66.0)	436 (58.9)	
Absent		586 (37.3)	282 (34.0)	304 (41.1)	
CVD	1,570				< .001
Present		119 (7.6)	44 (5.3)	75 (10.1)	
Absent		1,451 (92.4)	786 (94.7)	665 (89.9)	
Anemia	1570				< .001
Present		72 (4.6)	52 (6.3)	20 (2.7)	
Absent		1,498 (95.4)	778 (93.7)	720 (97.3)	
Hypothyroidism	1,570				.061
Present		247 (15.7)	117 (14.1)	130 (17.6)	
Absent		1,323 (84.3)	713 (85.9)	610 (82.4)	
MCI	1,570				.001
Present		219 (13.9)	139 (16.7)	80 (10.8)	
Absent		1,351 (86.1)	691 (83.3)	660 (89.2)	
Dementia	1,570				.033
Present		94 (6.0)	60 (7.2)	34 (4.6)	
Absent		1,476 (94.0)	770 (92.8)	706 (95.4)	
Glucose	1,563	106.8 (30.0)	113.5 (36.6)	99.2 (17.0)	< .001
Type 2 diabetes	1,570				< .001
Present		375 (23.9)	293 (35.3)	82 (11.1)	
Absent		1,195 (76.1)	537 (64.7)	658 (88.9)	
HbA1c	1,561	5.94 (1.1)	6.33 (1.4)	5.51 (0.6)	< .001
Aβ_42/40_ ratio	1,552	.049 (.012)	.051 (.012)	.047 (.011)	< .001
t-tau	1,570	2.45 (.93)	2.56 (.95)	2.33 (.90)	< .001
NfL	1,553	18.2 (10.1)	16.3 (9.5)	20.3 (10.3)	< .001

*Note*. MMSE = Mini-Mental State Examination; GDS = Geriatric Depression Scale, BMI = Body Mass Index; CVD = Cardiovascular disease, MCI = Mild cognitive impairment.

^a^ Is the *p* value for the Welch’s independent-samples *t* test, for numeric variables, or Fisher’s exact test, for categorical variables, assessing differences between Mexican Americans and Non-Hispanic Whites.

### Relationships of blood glucose, HbA1c, and T2D diagnosis with plasma Aβ_42/40_ ratio, t-tau, and NfL

No convergence problems were encountered with the MCMC estimation, as all values of the potential scale reduction factor were below 1.05 prior to the 500^th^ iteration of the burn-in phase. Table 2 shows the results for Aβ_42/40_ ratio. The model with the covariates and focal predictors (blood glucose, HbA1c, and T2D) accounted for 6%, Wald χ^2^(24) = 75.11, *p* < .001, of the variation in Aβ_42/40_ ratio, and the incremental variance due the focal predictors was < 1%, Wald χ^2^(3) = 0.65, *p* = .89. None of the focal predictors were significantly related to Aβ_42/40_ ratio. Among the covariates, Mexican American participants generally had greater values for Aβ_42/40_ ratio (*b* = .42, *p* < .001, *β* = .37) as did participants with *APOE*4 positivity (*b* = .23, *p* < .001, *β* = .20).

**Table 2:** Regression results for biomarker Aβ_42/40_ ratio (N = 1,552).

	Model 1			Model 2			Model 3		
Predictors	*b*	*SD*_*p*_	*β*	*b*	*SD*_*p*_	*β*	*b*	*SD*_*p*_	*β*
Intercept	3.767	.581	—	3.759	.580	—	3.886	.597	—
Ethnicity^b^	**.427** ^**^	.087	**.371**	**.421** ^***^	.088	**.366**	.332^**^	.1083	**.299**
Age	.006	.005	.045	.006	.005	.043	.005	.005	.041
Sex^c^	-.001	.070	-.001	-.002	.070	-.002	-.003	.070	-.003
Education (years)	< .001	.010	< .001	< .001	.010	< .001	.001	.010	.004
Married^d^	-.043	.068	-.037	-.043	.068	-.037	-.043	.068	-.037
Income^e^	.010	.007	.049	.010	.007	.050	.010	.007	.049
Homeowner^f^	-.054	.073	-.047	-.053	.073	-.046	-.052	.074	-.045
Years living in U.S.	< .001	.002	-.003	< .001	.002	-.004	< .001	.002	-.005
Smoke currently^g^	-.054	.129	-.047	-.049	.128	-.043	-.046	.130	-.040
*APOE*4 positivity^h^	**.232** ^***^	.069	**.200**	**.233** ^**^	.070	.203	**.232** ^**^	.070	**.202**
MMSE	.014	.015	.036	.014	.015	.037	.012	.015	.033
Health status	-.042	.036	-.038	-.041	.037	-.036	-.041	.037	-.036
GDS total	-.002	.006	-.008	-.001	.006	-.007	-.002	.006	-.008
BMI	.012	.010	.063	.012	.010	.062	.012	.010	.061
Ab circumference	-.007	.011	-.034	-.006	.011	-.031	-.007	.011	-.031
Hypertension^h^	.041	.063	.036	.042	.065	.037	.041	.064	.036
CVD^h^	-.084	.113	-.073	-.083	.113	-.072	-.089	.115	-.077
Anemia^h^	.072	.148	.063	.066	.149	.057	.069	.150	.060
Hypothyroidism^h^	.078	.082	.068	.073	.081	.063	.074	.082	.064
Cognitive disorder									
MCI vs. normal	-.014	.089	-.012	-.012	.088	-.010	-.011	.089	-.010
Dementia vs. normal	.022	.144	.019	.029	.143	.025	.028	.144	.024
Glucose	—	—	—	-.001	.002	-.035	-.002	.004	-.047
A1c	—	—	—	.031	.052	.030	.138	.124	.135
Diabetes^h^	—	—	—	.013	.100	.011	-.194	.174	-.169
Glucose × Ethnicity	—	—	—	—	—	—	.001	.004	.015
A1c × Ethnicity	—	—	—	—	—	—	-.136	.136	-.119
Diabetes × Ethnicity	—	—	—	—	—	—	.302	.212	.101
*R* ^*2*^	.060***	—	—	.061***	—	—	.065***	—	—
Δ *R*^*2*^	.060***	—	—	.001	—	—	.004	—	—

*Note*. Model 1 excluded the focal predictors and their interaction terms; Model 2 added the focal predictors; Model 3 added the set of two-way interactions.

*b* is a raw score regression coefficient. *SDp* is the standard deviation of posterior distribution. *β* is a standardized regression coefficient. MMSE = Mini-Mental State Examination; GDS = Geriatric Depression Scale, BMI = Body Mass Index; CVD = Cardiovascular disease, MCI = Mild cognitive impairment.

^a^ Values of Aβ_42/40_ ratio were multiplied by 100 to reduce the number of leading zeros.

^b^ Coded as 1 = Mexican-American; 0 = non-Hispanic white.

^c^ Coded as 1 = female; 0 = male.

^d^ Coded as 1 = married; 0 = not married.

^e^ Values of income were divided by 10,000 to reduce the number of leading zeros.

^f^ Coded as 1 = homeowner; 0 = otherwise.

^g^ Coded as 1 = yes; 0 = no.

^h^ Coded as 1 = condition is present, 0 = condition is absent.

*p < .05.

**p < .01.

***p < .001.

Table 3 shows the regression results for t-tau. The model with the same predictors accounted for 15%, Wald χ^2^(24) = 250.95, *p* < .001, of the variation, and the variance due uniquely to the focal predictors, less than 1%, was not statistically significant Wald χ^2^(3) = 6.78, *p* = .08. Participants diagnosed with T2D generally had greater values for t-tau (*b* = .16, *p* < .05, *β* = .17). For the covariates, participants who are Mexican Americans (*b* = .20, *p* < .01, *β* = .21), female (*b* = .26, *p* < .001, *β* = .28), unmarried (*b* = -.12, *p* < .05, *β* = -.13), with greater BMI (*b* = .04, *p* < .001, *β* = .22), and diagnosis of anemia (*b* = .78, *p* < .001, *β* = .84) had greater values for t-tau.

**Table 3:** Regression results for biomarker Tau (N = 1,570).

	Model 1			Model 2			Model 3		
Predictors	*b*	*SD*_*p*_	*β*	*b*	*SD*_*p*_	*β*	*b*	*SD*_*p*_	*β*
Intercept	.792	.442	—	.811	.454	—	.776	.456	—
Ethnicity^a^	**.211** ^**^	.066	**.223**	**.195** ^**^	.067	.**209**	.186^*^	.083	**.199**
Age	.006	.004	.060	.006	.004	.060	.007	.004	.061
Sex^b^	**.269** ^***^	.053	**.288**	**.263** ^***^	.054	**.282**	**.264** ^***^	.053	**.283**
Education (years)	.002	.008	.011	.003	.008	.014	.004	.008	.019
Married^c^	**-.121** ^*^	.052	**-.130**	**-.124** ^*^	.052	**-.133**	**-.121** ^*^	.052	**-.130**
Income^d^	-.009	.005	-.052	-.008	.005	-.051	-.009	.005	-.056
Homeowner^e^	-.014	.056	-.015	-.012	.056	-.013	-.016	.056	-.017
Years living in U.S.	.003	.002	.058	.002	.002	.053	.002	.002	.052
Smoke currently^f^	.046	.098	.049	.048	.100	.051	.050	.098	.054
*APOE*4 positivity^g^	.068	.054	.073	.062	.054	.066	.062	.053	.066
MMSE	.012	.011	.038	.012	.012	.038	.011	.012	.036
Health status	-.006	.028	-.007	.002	.028	.002	.002	.028	.002
GDS total	.003	.004	.020	.003	.004	.018	.003	.004	.016
BMI	.035^***^	.008	**.221**	.035^***^	.008	**.221**	.035^***^	.008	**.223**
Ab circumference	-.015	.008	-.093	-.016	.008	-.093	-.016	.008	-.093
Hypertension^g^	.084	.049	.090	.075	.049	.080	.076	.050	.081
CVD^g^	.112	.087	.120	.113	.086	.121	.110	.087	.118
Anemia^g^	**.827** ^***^	.108	**.886**	**.784** ^***^	.109	**.839**	**.784** ^***^	.110	**.840**
Hypothyroidism^g^	.068	.062	.073	.060	.063	.064	.064	.063	.069
Cognitive disorder									
MCI vs. normal	.013	.068	.014	.018	.068	.019	.022	.069	.024
Dementia vs. normal	.119	.110	.127	.127	.110	.136	.118	.110	.126
Glucose	—	—	—	-.002	.001	-.067	< .001	.003	< .001
A1c	—	—	—	.017	.038	.021	-.064	.095	-.078
Diabetes^g^	—	—	—	**.162** ^*^	.076	**.173**	.077	.135	.082
Glucose × Ethnicity	—	—	—	—	—	—	-.003	.003	-.075
A1c × Ethnicity	—	—	—	—	—	—	.088	.103	.096
Diabetes × Ethnicity	—	—	—	—	—	—	.142	.163	.059
*R* ^*2*^	.147**	—	—	.152***	—	—	.155***	—	—
Δ *R*^*2*^	.147***	—	—	.005	—	—	.003	—	—

*Note*. Model 1 excluded the focal predictors and their interaction terms; Model 2 added the focal predictors; Model 3 added the set of two-way interactions.

*b* is a raw score regression coefficient. *SDp* is the standard deviation of posterior distribution. *β* is a standardized regression coefficient. MMSE = Mini-Mental State Examination; GDS = Geriatric Depression Scale, BMI = Body Mass Index; CVD = Cardiovascular disease, MCI = Mild cognitive impairment.

^a^ Coded as 1 = Mexican American; 0 = non-Hispanic white.

^b^ Coded as 1 = female; 0 = male.

^c^ Coded as 1 = married; 0 = not married.

^d^ Values of income were divided by 10,000 to reduce the number of leading zeros.

^e^ Coded as 1 = homeowner; 0 = otherwise.

^f^ Coded as 1 = yes; 0 = no.

^g^ Coded as 1 = condition is present, 0 = condition is absent.

*p < .05.

**p < .01.

***p < .001.

Table 4 shows that for NfL, 36%, Wald χ^2^(24) = 803.15, *p* < .001, of the variation was accounted for by the model, and the variance due uniquely to the focal predictors, 3.7%, was statistically significant, Wald χ^2^(3) = 84.47, *p* < .001. A1c (*b* = 2.21, *p* < .001, *β* = 0.25) was positively related to NfL, as were several covariates, including age (*b* = 0.44, *p* < .001, *β* = 0.38), years living in the U.S. (*b* = 0.04, *p* < .05, *β* = 0.07), smoking status (*b* = 2.00, *p* < .05, *β* = 0.20), and anemia diagnosis (*b* = 6.83, *p* < .001, *β* = 0.68), whereas BMI (*b* = -0.17, *p <* .05, *β* = -0.10), and Ab circumference (*b* = -0.16, *p* < .05, *β* = -0.09), were negatively related to NfL. Mexican American participants generally had lower NfL values than non-Hispanic Whites (*b* = -2.23, *p* < .001, *β* = -0.22), whereas participants with mild cognitive impairment (*b* = 1.90, *p* < .01, *β* = 0.19) or dementia (*b* = 2.63, *p* < .05, *β* = 0.26), had greater NfL values compared to those with normal cognition.

**Table 4:** Regression results for biomarker Nfl (N = 1,553).

	Model 1	Model 2	Model 3
Predictors	*b*	*SD*_*p*_	*β*	*b*	*SD*_*p*_	*β*	*b*	*SD*_*p*_	*β*
Intercept	-6.410	4.326	—	-3.219	4.228	—	-6.898	4.261	—
Ethnicity^a^	-1.146	.640	-.114	**-2.267** ^***^	.638	**-.223**	-.720	.766	-.072
Age	**.456** ^***^	.034	.**398**	**.442** ^***^	.034	**.384**	**.449** ^***^	.034	.**391**
Sex^b^	-.179	.517	-.018	-.392	.503	-.039	-.465	.502	-.046
Education (years)	.092	.074	.044	.089	.073	.043	.096	.072	.046
Married^c^	-.244	.501	-.024	-.331	.492	-.033	-.388	.486	-.039
Income^d^	-.045	.050	-.026	-.032	.049	-.018	-.041	.049	-.023
Homeowner^e^	-.797	.542	-.079	-.627	.533	-.062	-.658	.525	-.065
Years living in U.S.	**.038** ^*^	.018	**.077**	.036*	.017	**.074**	.034*	.017	**.068**
Smoke currently^f^	**2.127** ^*^	.969	**.212**	**1.998** ^*^	.929	**.199**	**2.079** ^*^	.926	**.207**
*APOE*4 positivity^g^	.067	.515	.007	.258	.504	.026	.207	.506	.036
MMSE	-.078	.113	-.024	-.007	.109	-.002	.001	.109	.021
Health status	-.527	.269	-.054	-.216	.266	-.022	-.274	.263	-.028
GDS total	.058	.042	.033	.064	.041	.037	.060	.041	.035
BMI	**-.177** ^*^	.075	**-.104**	**-.166** ^*^	.073	**-.097**	**-.160** ^*^	.073	**-.094**
Ab circumference	-.080	.081	-.044	**-.156** ^*^	.079	**-.087**	-.125	.079	-.069
Hypertension^g^	**1.146** ^*^	.476	**.114**	.804	.467	.080	.952	.463	.095
CVD^g^	-.208	.838	-.021	-.146	.819	-.015	.079	.807	.008
Anemia^g^	**7.456** ^***^	1.082	**.742**	**6.826** ^***^	1.074	.**679**	**6.614** ^***^	1.058	**.658**
Hypothyroidism^g^	.348	.602	.035	.211	.583	.021	.230	.579	.023
Cognitive disorder									
MCI vs. normal	**1.774** ^**^	.661	**.176**	**1.896** ^**^	.639	**.189**	**1.805** ^**^	.629	**.180**
Dementia vs. normal	2.055	1.070	.204	**2.630** ^*^	1.056	**.262**	**2.536** ^*^	1.047	**.252**
Glucose	—	—	—	-.024	0.012	-.007	**-.087** ^**^	.027	**-.259**
A1c	—	—	—	**2.205** ^***^	.378	**.247**	.345	.893	.039
Diabetes^g^	—	—	—	.846	.730	.084	**3.321** ^**^	1.263	**.331**
Glucose × Ethnicity	—	—	—	—	—	—	**0.079** ^*^	0.030	**.209**
A1c × Ethnicity	—	—	—	—	—	—	**1.964** ^*^	.976	**.195**
Diabetes × Ethnicity	—	—	—	—	—	—	**-3.079** ^*^	1.525	**-.119**
*R* ^*2*^	.321***	—	—	.358***	—	—	.371***	—	—
Δ *R*^*2*^	.321***	—	—	.037***	—	—	.013***	—	—

*Note*. Model 1 excluded the focal predictors and their interaction terms; Model 2 added the focal predictors; Model 3 added the set of two-way interactions.

*b* is a raw score regression coefficient. *SDp* is the standard deviation of posterior distribution. *β* is a standardized regression coefficient. MMSE = Mini-Mental State Examination; GDS = Geriatric Depression Scale, BMI = Body Mass Index; CVD = Cardiovascular disease, MCI = Mild cognitive impairment.

^a^ Coded as 1 = Mexican-American; 0 = non-Hispanic white.

^b^ Coded as 1 = female; 0 = male.

^c^ Coded as 1 = married; 0 = not married.

^d^ Values of income were divided by 10,000 to reduce the number of leading zeros.

^e^ Coded as 1 = homeowner; 0 = otherwise.

^f^ Coded as 1 = yes; 0 = no.

^g^ Coded as 1 = condition is present, 0 = condition is absent.

*p < .05.

**p < .01.

***p < .001.

### T2D and AD biomarker relationships by ethnicity

For Aβ_42/40_ ratio, Table 2 shows that the set of interactions accounted for less than 1% of the variance, which was not significant, Wald χ^2^(3) = 2.21, *p* = .53. None of the specific two-way interactions were significant (each *p* > .15). Similarly, for t-tau, Table 3 shows that the set of interactions accounted for less than 1% of the variance, which was not significant, Wald χ^2^(3) = 2.68, *p* = .44, and that none of the specific interactions were significant (each *p* > .38).

**Fig 1:**
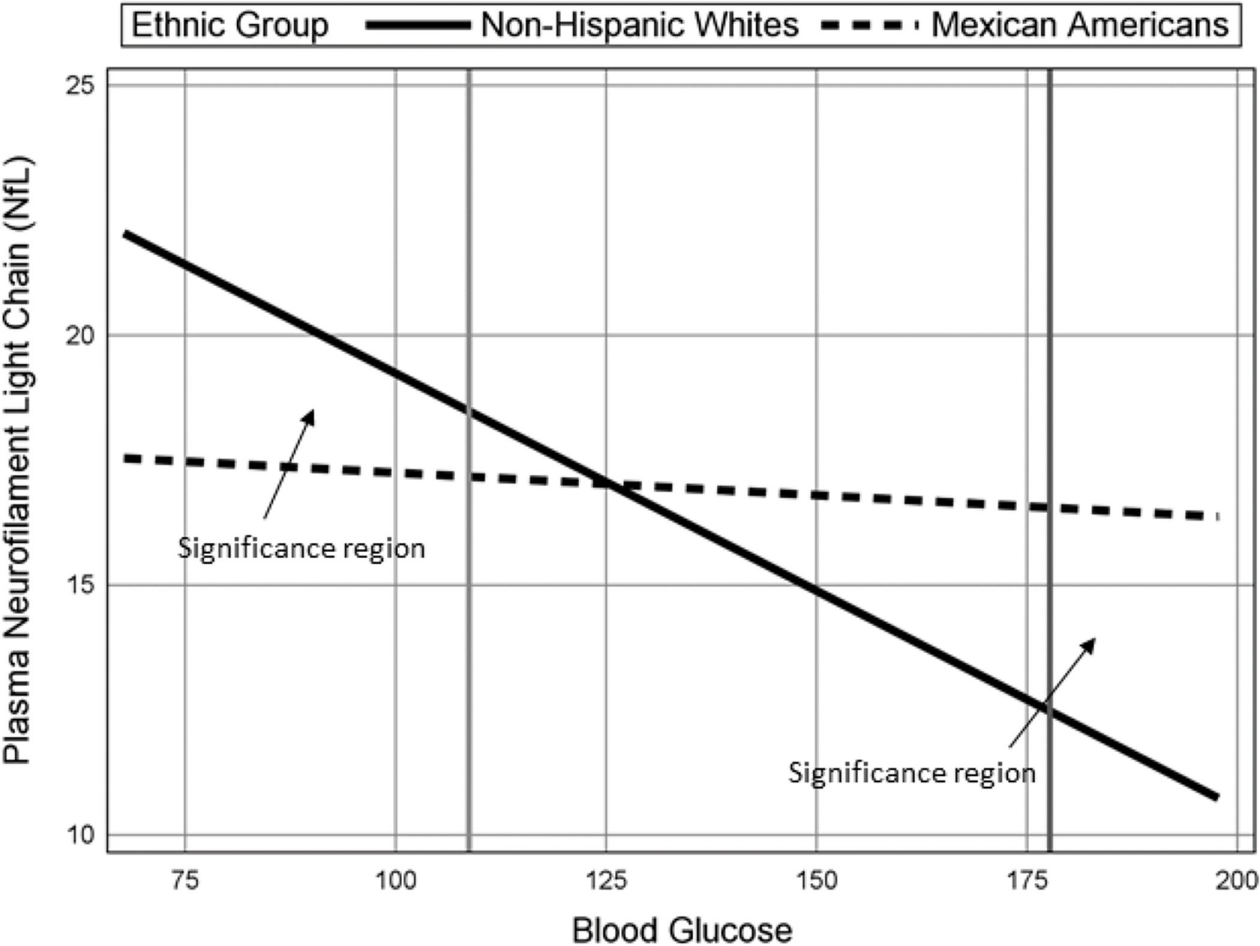
Plasma neurofilament light change with blood glucose by ethnic group. All Other Predictors Held Constant at their Mean.

For NfL, Table 4 shows that the set of interactions accounted for an additional 1.3% of variance, Wald χ^2^(3) = 29.77, *p* < .001, and that each two-way interaction was significant (each *p* value < .05). Fig 1 displays the plot of the glucose-by-ethnicity interaction for blood glucose that are common to each ethnic group and shows that the association between blood glucose and NfL, as represented by the slope of the lines, is negative for non-Hispanic Whites (*b =* -0.09, *p* < .01, *β =* -0.26) but not for Mexican American participants (*b* = -.009, *p* = .51, *β =* -0.03). Further, the significance regions shown in Fig 1 indicate that non-Hispanic Whites have significantly greater NfL values when glucose is lower than 109, whereas Mexican American participants have significantly greater NfL when glucose is greater than approximately 178.

**Fig 2:**
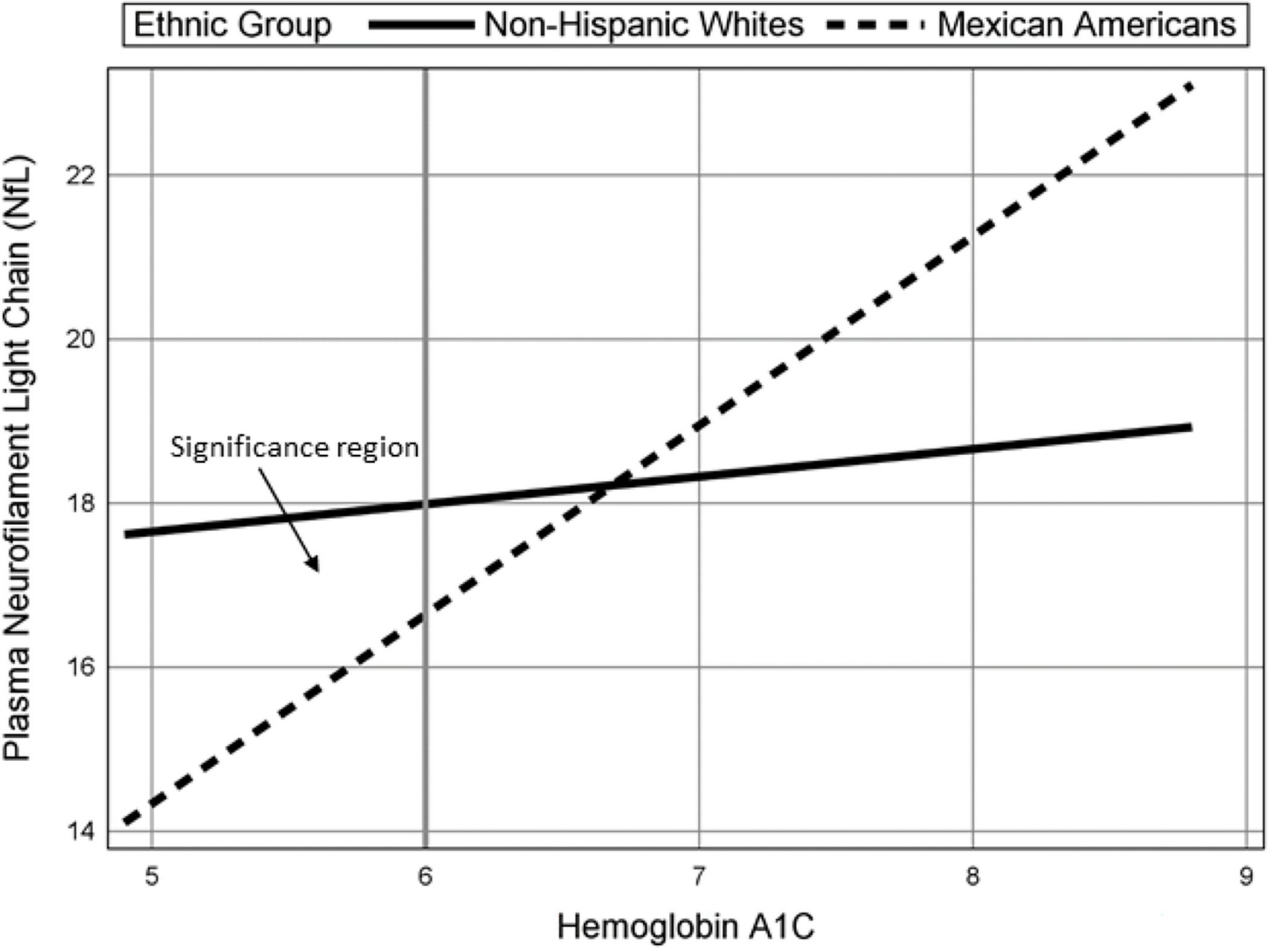
Plasma neurofilament light change with hemoglobin A1c by ethnic group. All Other Predictors Held Constant at their Mean.

Fig 2 displays the plot of the HbA1c-by-ethnicity interaction for HbA1c values that are common to each ethnic group and shows that HbA1c is not related to NfL for non-Hispanic Whites (*b =* 0.34, *p* = .71, *β =* 0.04) but is positively related to NfL for Mexican American participants (*b* = 2.31, *p* < .001, *β =* 0.26). Further, the significance region shown in Fig 2 indicates that non-Hispanic Whites have significantly greater NfL values than Mexican American participants when A1c is below a value of 6.0.

**Fig 3:**
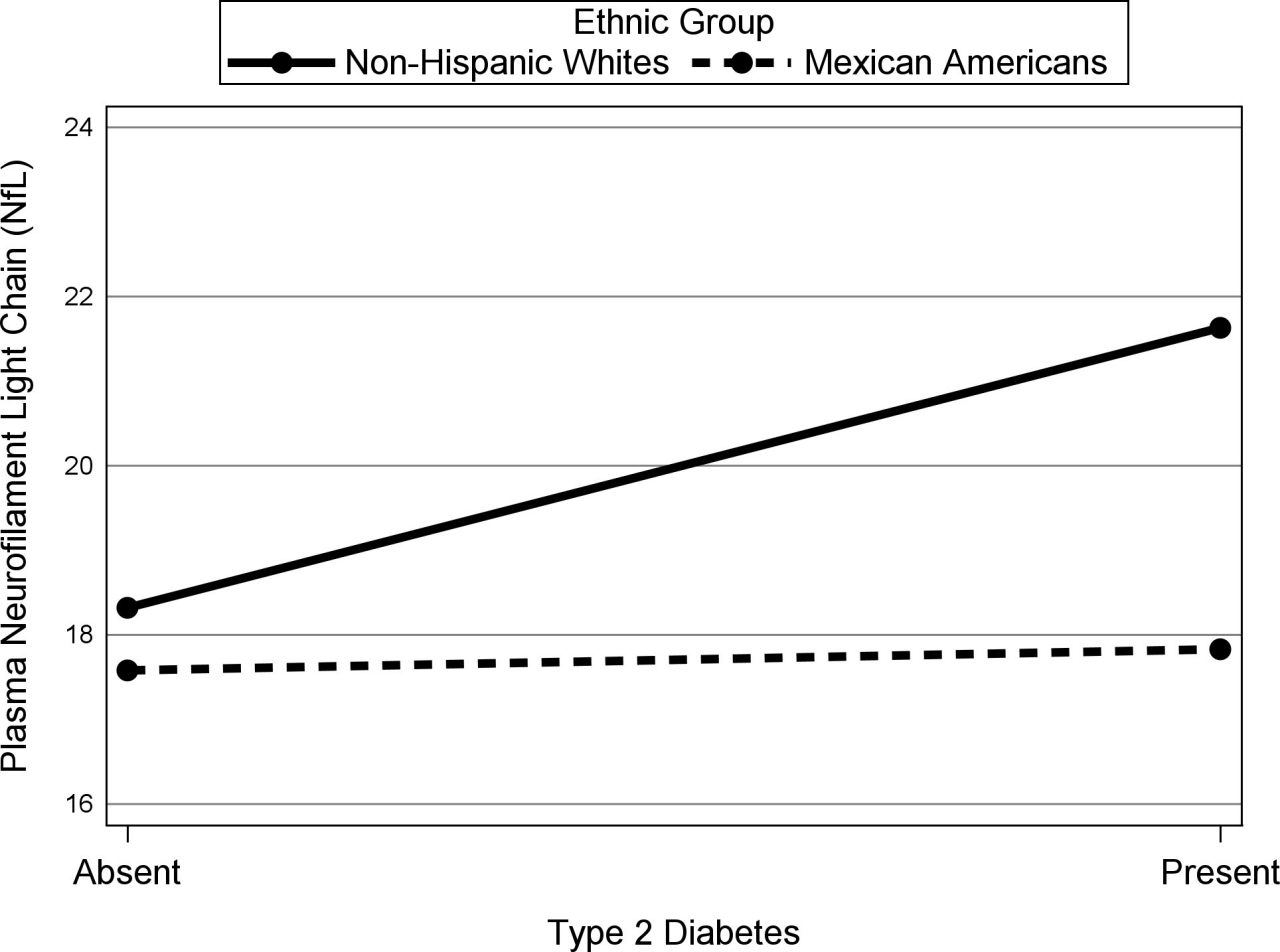
Plasma neurofilament light change with type 2 diabetes diagnosis by ethnic group. All Other Predictors Held Constant at their Mean.

Fig 3 displays a plot of the interaction between T2D and ethnicity and shows that for non-Hispanic Whites, participants diagnosed with T2D have greater NfL values than those without T2D (*b* = 3.32, *p* < .01, *β =* 0.33) whereas T2D is not related to NfL for Mexican American participants (*b* = 0.25, *p* = .77, *β =* 0.02). In addition, for participants without T2D, predicted NfL values do not differ by ethnicity (*b* = -0.72, *p* = .34, *β =* -0.07). However, for participants with T2D, non-Hispanic Whites have greater NfL values compared to Mexican Americans (*b* = 3.81, *p* < .01, *β =* 0.38). Note that including the significant interactions in the model for hypothesis 2 resulted in trivial changes to the regression results reported for hypothesis 1, except that Ab circumference (*b* = -0.13, *p* = .11, *β =* -0.07) is no longer related to NfL.

## Discussion

The main findings from our study showed that blood glucose, blood HbA1c, and T2D diagnosis explained 3.7% of the variation in NfL but were not related to Aβ_42/40_ ratio and t-tau. HbA1c was positively associated with NfL among Mexican Americans, but blood glucose and T2D diagnosis were not associated with NfL. In contrast, blood glucose was negatively associated with NfL, HbA1c was not associated with NfL, and T2D diagnosis was positively associated with NfL among non-Hispanic Whites.

Few studies have examined the relationships of blood glucose, blood HbA1c, and T2D diagnosis with AD plasma biomarkers. Our study showed that blood glucose, blood HbA1c, and T2D diagnosis explained 3.7% of the variation in NfL but were not related to Aβ_42/40_ ratio and t-tau, suggesting that T2D may not play a role in Aβ accumulation but may be more important to neurodegeneration. NfL indicates subcortical large-caliber axonal degeneration [58, 59]. Elevated plasma NfL levels have been established in AD [60, 61], correlate to increasing symptom severity in AD [62], and predicts greater long-term cognitive decline in AD [63–65]. The positive association between the three T2D indicators and plasma NfL is consistent with existing evidence that used imaging biomarkers of neurodegeneration [30, 35, 36]. Together, the current literature and our findings indicate that T2D may contribute to AD pathogenesis through neurodegeneration, particularly among Mexican Americans. Hence, assessing neurodegeneration among Mexican Americans with pre-T2D and T2D is critical for identifying early signs of neurodegeneration. Early diagnosis and management of T2D may play an important role in slowing down the progression of AD.

Our findings further show that higher HbA1c levels were not associated with Aβ_42/40_ ratio and t-tau but were associated with higher plasma NfL levels. Our findings support the previously reported lack of association between HbA1c with CSF Aβ_42_ [30]. Our study used the more sensitive Aβ_42/40_ ratio than Aβ_42_ or Aβ_40_ concentrations because it normalizes inter-individual differences in Aβ production as a more sensitive measure [41]. Moreover, our study showed higher plasma Aβ_42/40_ ratio in Mexican Americans than in non-Hispanic Whites, indicating less Aβ burden, but did not identify ethnic difference in the relationships between HbA1c and plasma Aβ_42/40_. Since decreased plasma Aβ_42/40_ ratio is believed to reflect higher Aβ load in the brain [66], it will be important to further examine if cerebral Aβ load differs between Mexican Americans and non-Hispanic Whites and whether higher plasma Aβ_42/40_ ratio is associated with better cognition and lower risk of dementia in Mexican Americans.

We further found the association between HbA1c and NfL, but not between blood glucose or T2D diagnosis and NfL among Mexican Americans only. These findings indicate that adequate, chronic control of T2D may be particularly beneficial for mitigating neurodegeneration in Mexican Americans, which needs to be further tested. Both T2D and dementia diagnoses were more common in our Mexican American cohort than the non-Hispanic White cohort, which is consistent with previous reports of the disproportionate impacts of both conditions in Hispanic Americans [44–47]. The literature on the associations of T2D diagnosis with cognitive impairment and AD have been mixed [19, 30, 37–39]. Some studies did not find an association of T2D with CSF Aβ_42_ [30] or memory in individuals with AD [37]. Some suggested that T2D diagnosis was associated with higher plasma levels of Aβ_42_, Aβ_40,_ and t-tau among cognitively unimpaired older adults [67]. Others reported that T2D might be cognitively and functionally protective in older adults with mild AD dementia [38, 39]. When analyzing HABS-HD participants with normal cognition (n = 965), a diagnosis of T2D was significantly associated with plasma Aβ_42,_ Aβ_42,_ t-tau, and NfL [50]. However, we found no associations of T2D diagnosis with any of the ATN biomarkers. Together, these findings indicate that the association between T2D diagnosis and ATN biomarkers may vary by populations and T2D pathologic burden as reflected by glycemic control and diabetic complications may be more important for understanding the role that T2D plays in AD [19].

Plasma t-tau may reflect Aβ-induced tau secretion in AD [41]. but it is currently considered a neurodegeneration biomarker [68]. Our study showed that none of the focal predictors significantly predicted plasma t-tau. Furthermore, our study did not find any differences in the relationships of blood glucose, blood HbA1c, and T2D diagnosis with plasma t-tau between Mexican Americans and non-Hispanic Whites. These findings may be explained by the lack of understanding of the role of plasma t-tau in AD [67]. Future studies are needed to examine if the relationships of blood glucose, HbA1c, and T2D diagnosis with plasma phosphorylated tau exist and whether these relationships are moderated by ethnicity.

There is evidence that impaired fasting glucose is associated with increased cerebral Aβ burden [32] and accelerates AD clinical progression [19, 33]. In this study. plasma Aβ_42/40_ ratio and t-tau levels were higher in Mexican Americans than in non-Hispanic Whites, which are consistent with a previous analysis of HABS-HD participants with normal cognition (n = 965) [50]. Moreover, we found that fasting glucose was negatively associated with plasma NfL level among non-Hispanic Whites, but not among Mexican Americans. Our findings may be influenced by other factors which could have affected plasma AD biomarker levels. For example, kidney function was found to attenuate the association between intensive hypertension treatment and NfL [69]. In our study, estimated glomerular filtration rate was a significant negative predictor (r = -0.30) for plasma NfL levels. However, including estimated glomerular filtration rate did not change the study results on NfL. These findings suggest that plasma biomarkers need to be interpreted within the context of ethnicity and the importance of developing ethnicity-specific normative biomarker values to guide clinical practice and future research [50].

The strengths of this study included a large representative sample of Mexican Americans and non-Hispanic Whites and rigorous methods in data collections and blood processing following established protocols in the HABS-HD. We were able to examine three clinical indicators of T2D and AD plasma biomarkers, respectively, which are all highly scalable clinical measures, while controlling for a range of covariates which may affect AD biomarker levels. Our study was limited by its cross-sectional design and the lack of measures of phosphorylated tau. About 25% of HABS-HD participants were excluded due to missing data or as large outliers, which may have affected the study results. Our findings need to be further validated in other cohorts and longitudinally.

## Conclusions

This study found that blood glucose, blood HbA1c, and T2D diagnosis may contribute to neurodegeneration, but probably not Aβ. Fasting blood glucose and T2D diagnosis were associated with NfL among non-Hispanic Whites, while HbA1c was associated with NfL among Mexican Americans. These findings add to the existing evidence about the pathological cross-link between T2D and AD. This preliminary cross-sectional observation needs to be confirmed by a prospective cohort study.
